# Novel cubosome based system for ocular delivery of acetazolamide

**DOI:** 10.1080/10717544.2021.1989090

**Published:** 2021-10-18

**Authors:** Hoda E. Teba, Islam A. Khalil, Heba M. El Sorogy

**Affiliations:** Department of Pharmaceutics, Faculty of Pharmacy and Drug Manufacturing, Misr University for Science and Technology, 6th of October, Egypt

**Keywords:** Glaucoma, intraocular pressure, acetazolamide, carbonic anhydrase inhibitor, cubosomes, ocular drug delivery

## Abstract

Acetazolamide is the drug of choice for glaucoma treatment in an emergency. However, it is not available in any topical formulation and it is available only as systemic tablets. Despite its efficiency as a drug in decreasing intraocular pressure, it has negative systemic effects as renal toxicity and metabolic acidosis. Moreover, it suffers from poor aqueous solubility and low corneal permeability limiting its ocular bioavailability and its use topically. Cubosomes have enormous advantages as a drug delivery system, most importantly, high surface area, thermal stability, and ability to encapsulate hydrophobic, amhiphilic, and hydrophilic molecules. Herein, we have exploited the unique properties of cubosomes as a novel nano-delivery system for acetazolamide as eye drops dosage form for glaucoma treatment. Different acetazolamide-loaded cubosomes have been developed and evaluated. The best-optimized formulation (F5), was cubic shaped structure, with an average particle size of 359.5 ± 2.8 nm, surface charge −10.8 ± 3.2 mV, and 59.8% entrapment efficiency. *Ex-vivo* corneal permeation studies have revealed a 4-fold increase in acetazolamide permeability coefficient compared to that stated in the literature. F5 showed superior therapeutic efficacy represented by a 38.22% maximum decrease in intraocular pressure *vs.* 31.14 and 21.99% decrease for the commercial Azopt^®^ eye drops and Cidamex^®^ tablets, respectively. It also exhibited higher (AUC_0–10_) compared to Azopt^®^ eye drops and Cidamex^®^ tablets by 2.3 and 3 times, respectively. F5 showed mean residence time 4.22 h *vs.* 2.36 and 2.62 h for Azopt^®^ and Cidamex^®^ with no eye irritation observed according to the modified Draize test. To the best of our knowledge, this is the first study for developing acetazolamide-loaded cubosomes as the topical delivery system for glaucoma treatment.

## 1. Introduction

Glaucoma is a major global health problem, with about 80 million glaucomatic individuals worldwide in 2020 (Allison et al., 2020). It acts as the second cause of irreversible blindness (Occhiutto et al., 2020). Glaucoma is a neurodegenerative eye disorder causing elevated intraocular pressure (IOP) leading to gradual retinal ganglion cells degeneration, optic nerve atrophy, and irreversible blindness (Cantor, 2006). Elevated IOP is caused predominantly by blockage of the outflow system. Therefore, the goal of all glaucoma therapies is to reduce IOP by either suppressing production or enhancing trabecular meshwork and uveoscleral outflow of aqueous humor (Heijl et al., 2002).

Acetazolamide (ACZ) is used in the treatment of glaucoma, with the aim of lowering IOP, especially in emergency cases. It exerts its action by inhibiting the activity of carbonic anhydrase in the ciliary processes of the eye and consequently decreases the production of the aqueous humor. Despite its potent action in glaucoma management, a high oral dose (500 mg) is needed to achieve its therapeutic effect by carbonic anhydrase inhibition in the peripheral system. Subsequently, the broad range of systemic side effects is associated with ACZ that interfere with patient convenience (Morsi et al., 2017). The most common side effects are diuresis, renal failure, vomiting, anorexia, central nervous system depression, and metabolic acidosis (Granero et al., 2008; Mahmoud et al., 2019).

The topical formulations for ACZ are not available in the market until now as it has poor solubility in water and limited corneal permeability which resulted in low ocular bioavailability, causing an inadequate amount of the drug to reach the ciliary body. Numerous studies tried to improve ACZ ocular bioavailability to develop an effective topical formulation. Among these studies is the formulation of ACZ in aqueous solutions containing cyclodextrins or penetration enhancers and polymeric suspension (Loftsson et al., 1994; Kaur & Smitha, 2002). Other studies employed nanotechnology-based approaches through vesicular preparations as niosomal and liposomal dispersions (Guinedi et al., 2005; Hathout et al., 2007), modified vesicles (Naguib et al., 2017), nanoparticles (Singh et al., 2014; Rathod et al., 2017), dendrimers (Mishra & Jain, 2014; Bravo-Osuna et al., 2016), and nanoemulsions (Morsi et al., 2014; Morsi et al., 2017).

Cubosomes have gained growing attention as ophthalmic nanocarriers in the last few years (Verma & Ahuja, 2016; Huang et al., 2017) due to their biocompatibilities and bioadhesive properties (Matloub et al., 2018). In addition, cubosomes are characterized by their high loading capacity for many drugs of different hydrophilicities. They also enable targeted and controlled drug release, enhance drug stability, improve transcorneal permeability, prolong corneal retention, and are prepared by simple techniques at low cost (Gan et al., 2010; Chen et al., 2014; Younes et al., 2018).

Cubosomes have a unique structure formed by dispersion of self-assembled amphiphilic lipid molecules as a liquid crystalline phase with cubic crystallographic symmetry in aqueous media (Scriven, 1976). They are characterized by high surface area due to the presence of two continuous water channels separated by a contorted lipid bilayer (Younes et al., 2018). They have a structure similar to honeycomb (cavernous) structures with a size range of 100–500 nm (Esposito et al., 2005; Huang et al., 2017). Due to the previously mentioned properties of cubosomes, it was selected as a delivery system for ACZ as it would be expected to overcome the main problems facing the preparation of ACZ as topical eye drops by improving both drug solubility and permeation.

The objective of this study was to formulate a topical ACZ-loaded cubosomal delivery system for management of glaucoma aiming to increase drug penetration across the cornea, provide the advantage of eye drops, perform targeted inhibition of carbonic anhydrase enzyme within the eye to reduce systemic side effects of ACZ and improve patient compliance.

## 2. Materials and methods

### 2.1. Materials

Acetazolamide (ACZ) (99.9% purity) was obtained from CID Company, Egypt. Transcutol^®^ P (diethylene glycol monoethyl ether) and Peceol^®^ TM (glyceryl monooleate) (GMO) were kindly donated by Gattefosse, France. Pluronic^®^ F127 (Poloxamer 407) (P407) was purchased from BASF Germany. Propylene glycol (PG), potassium dihydrogen phosphate, disodium hydrogen phosphate, sodium bicarbonate, calcium chloride, and sodium chloride were purchased from El-Nasr Pharmaceutical Chemicals Co., Egypt.

### 2.2. Preparation of ACZ-loaded cubosomes

Cubosome dispersions were prepared by emulsification technique (Elgindy et al., 2016). The lipids phase (3.85–6.25% w/w of total dispersion), consist of GMO and P 407 at different ratios [2:1, 4:1, and 6:1], were melted at 60 °C (heating magnetic stirrer, Thermolyne Corp.). Then, ACZ was added to the molten oily phase and solubilized under stirring. Afterward, PG or Transcutol^®^ P (2.5 and 10% w/w) were dissolved in water and heated to the same temperature and then was added to the molten mixture with stirring at 500 rpm. The formed dispersions were kept under stirring for 2 h at room temperature to allow solidification of the lipid droplets. Dispersions were then homogenized at 15,000 rpm and 60 °C for 1 min (Heidolph Homogenizers, Silent Crusher M, Germany). After cooling, the dispersions were maintained at room temperature in glass vials. The composition of various cubosmes dispersions were shown in Table 1.

**Table 1.** Composition of various formulations of cubosomes dispersions.

Formulation	GMO: P407	Oil: water	PG % (w/w)	Transcutol^®^ P % (w/w)	ACZ (mg)
F1	2:1	1:20	2.5	…	200
F2	4:1	1:20	2.5	…	200
F3	6:1	1:20	2.5	…	200
F4	2:1	1:15	2.5	…	200
F5	2:1	1:25	2.5	…	200
F6	2:1	1:25	10	…	200
F7	2:1	1:25	…	2.5	200
F8	2:1	1:25	…	10	200

### 2.3. Characterization of ACZ-loaded cubosomes

#### 2.3.1. Determination of particle size, polydispersity index (PDI), and zeta potential for ACZ-loaded cubosomes

The average particle size of the cubosomes and their size distribution (polydispersity index; PDI) were measured by a particle analyzer (Zetasizer Nano ZS, Ver. 6.20, Malvern Instruments Ltd., UK) on the base of dynamic light scattering at an angle of 173. Detection of zeta potential values of cubosomes depends on the laser Doppler anemometry of the Zetasizer instrument. All measurements were performed in triplicates at 25 °C.

#### 2.3.2. Determination of entrapment efficiency (E.E%) for cubosomes

The prepared ACZ-loaded cubosomes dispersions were centrifuged at 13500 rpm and 4 °C for 60 min (cooling ultracentrifuge 3–30 K, Sigma, Germany). Then, the supernatant was collected to determine the amount of free (non-entrapped) ACZ by spectrophotometric analysis (UV spectrophotometer; Shimadzu, USA) at 263 nm. Blanks were prepared by centrifuging plain cubosomes dispersions contain the same concentrations of all ingredients except ACZ. The entrapment percentage of ACZ in the cubosomes was calculated by subtracting the amount of free ACZ from the total amount of ACZ used to prepare the cubosomes. All measurements were performed in triplicates.

The entrapment efficiency (E.E%) was estimated according to the following equation:

(1) E.E %=[(total drug – free drug)/total drug]×100

#### 2.3.3. Determination of pH

The pH values of the prepared ACZ-loaded cubosomes nano-dispersions were determined by pH meters (Hanna, type 211, Romania).

### 2.4. Ex-vivo corneal permeation studies

The permeation of ACZ was determined through the corneal membrane obtained from goats. The corneas were carefully separated from the whole eyeballs with 3–4 mm of the surrounding scleral tissue. Corneas were washed with cold saline then preserved in a cold freshly prepared solution of simulated tear fluid (STF) of pH 7.4 (Gupta et al., 2007). The study was performed in triplicate using USP dissolution apparatus (Hanson RS8-plus, US). Cylindrical glass tubes of 0.5 cm diameters were fitted with goat corneas with epidermis directed toward the inner part of the tube where formulations were placed. The surface area of the cornea that is available for diffusion was 0.785 cm^2^. The receptor compartment was filled with STF pH 7.4 adjusted at 35 ± 0.5 °C and was stirred at 50 rpm. Periodically, samples were withdrawn at specified time intervals and replaced immediately with an equal volume of STF. Permeation study was carried out for 6 h. Withdrawn samples were analyzed spectrophotometrically (UV spectrophotometer; Shimadzu, USA) for the amount of ACZ permeated across the corneal membrane.

Amount ACZ permeated per unit area (µg·cm^−2^) was plotted *vs.* time (h). Permeation parameters, namely, steady-state flux, *J* (µg·cm^−2^ h^−1^) and permeation coefficient, *P* (cm·s^−1^) were calculated from slopes of linear ascents of permeation curves (Muchtar et al., 1997; Yang & Benita, 2000; Hegde et al., 2014) as follows:

(2) $$J\;=\;\mathrm{dQ}/\mathrm{dt}.\;A\;(\mu g.cm^{-2}h^{-1})$$

(3) $$P\;=\;J/(C_{\circ }\;\times \;60\;\times \;60)\;(\text{cm}.s^{-1})$$

where; *dQ*/*dt* represent slopes of linear ascents of permeation plots (µg·h^−1^), *A* is the corneal surface area available for diffusion (cm^2^) and *C_o_* is the initial drug concentration in donor compartment (µg·cm^−3^). Lag time, the time needed by the drug to saturate cornea and to reach to receptor compartment was calculated from *X*-axis intercept values of regression lines.

Corneal hydration was determined by weighting each cornea that was freed from adhering sclera, then was soaked in 1 ml methanol, dried overnight at 80 °C, and reweighed. Corneal hydration is calculated from the difference in weights (Motwani et al., 2008; Yadav & Ahuja, 2010; Mudgil & Pawar, 2013).

### 2.5. Selection of the optimum formulation

Optimum cubosomes formulation was selected for further studies depending on the previously performed characterization and *ex-vivo* permeation study. Small particle size, low PDI and high zeta potential, E.E%, and permeation parameters are the required characteristics in the optimum formula.

### 2.6. Morphology of the optimum ACZ-loaded cubosomes formulation

The morphological examination was performed using a transmission electron microscope, TEM (JOEL-JEM-1230, Japan). One drop of the selected optimum formulation (F5) was placed onto a copper grid and stained with 2%w/v phosphotungstic acid for 3 min before the examination.

### 2.7. Effect of gamma sterilization

F5 was subjected to Gamma irradiation in a screw-capped glass bottle at a dose of 25 kilos Gray (k Gy) at a rate of 1.323 k Gy/h using ^60^Co as a source of irradiation (Younes et al., 2018) at the National Center for Radiation Research and Technology (NCRRT), Cairo, Egypt. To study the effect of Gamma irradiation, F5 was then reexamined for particle size, PDI, zeta potential, and E.E% and compared to those measured before sterilization.

### 2.8. Ocular irritation studies

Evaluation of ocular irritancy of the optimized formulation (F5) was studied using adult male New Zealand albino rabbits (2–3 kg). The protocol of the study was approved by the Research Ethics Committee in the Faculty of Pharmacy, Misr University of Science and Technology, Egypt. The test was performed according to Modified Draize Test (Baeyens et al., 2002; Mishra & Jain, 2014). All glassware used in the experiments was sterilized by autoclaving and F5 was sterilized by Gamma irradiation.

Six New Zealand albino rabbits were divided into two groups of three rabbits each. The first group received normal saline and served as control and the second received F5. One drop (25 µl) of either F5 or normal saline was instilled into the lower cul-de-sac of the right eye of each rabbit using a needleless syringe. The untreated contralateral eye was used as a control.

The eyelids were held gently for 10 s to avoid loss of instilled formulation. Each animal was observed for ocular reactions (redness, discharge, and conjunctival chemosis) at 5, 10, 15, and 30 min and 1, 2, 3, 6, 9, 12, and 24 h post-administration. The ocular irritation was evaluated by using the clinical evaluation scale described in Table 2 (Mishra & Jain, 2014). Overall ocular irritation index was calculated from the clinical evaluation scores observed for each parameter at any specified time. If the score was 2 or 3 in any category or more than 4 in the overall ocular irritation index, it was indicated as significant irritation.

**Table 2.** Modified Draize grading scale for clinical evaluation of ocular irritation.

Score	Signs of conjunctival irritation		
	Discharge	Chemosis	Redness
0	Normal	Normal	Normal blood vessels
1	Slight discharge	Slight chemosis of nictating membrane	Some defined hyperemic blood vessels.
2	Severe discharge covering a small area around the cornea	Severe with partially closed eye	Diffuse color with vessels not easily distinguished
3	Severe discharge covering a large area around the cornea	Severe with completely closed eye	Diffuse beefy red

### 2.9. Therapeutic efficiency study

The study was approved by the ethics committee, Faculty of pharmacy, MUST University. Nine New Zealand albino normotensive rabbits (2–3 kg) were divided randomly into three groups (three rabbits in each group). IOP of the rabbits was measured using an indention tonometer (Schoetz tonometer, Germany). 1–2 drops of benoxinate hydrochloride (Benox^®^) eye drops were instilled into the rabbit eyes before measuring IOP for anesthesia. A normal baseline IOP was established for each animal by measuring the resting IOP twice a day one day before the experiment.

A single 50 µl dose of 0.8% w/w ACZ-loaded cubosome (F5) was applied topically to the cornea of the first group. In addition, a 50 µl dose of Azopt^®^ (brinzolamide, 1% w/v) was administered to the rabbits of the second group. The third group received orally a fraction of ACZ tablets (Cidamex^®^) equivalent to 9 mg ACZ. The IOP was measured for a period of 10 h after administration.

The percentage decrease in IOP was determined according to the following equation (Ammar et al., 2009):

(4) % Decrease in IOP = (Baseline IOP – IOP after treatment) × 100/baseline IOP.

Therapeutic profiles of F5, ACZ tablets (Cidamex^®^), and 1% brinzolamide eye drops (Azopt^®^) were obtained by plotting the percentage reduction of IOP *vs.* time. These profiles were used to calculate various pharmacodynamic parameters: area under the curve (AUC_0–10_), the time required to achieve maximum IOP reduction (*T*_max_), the maximum percentage decrease in IOP, and the mean residence time (MRT) using WinNonlin^®^ software (Ver. 1.5, Scientific consulting Inc., Cary, NC, USA).

Statistical analysis of the results was performed using one-way analysis of variance (ANOVA) which was computed using GraphPad Instat software (GraphPad Software, USA).

## 3. Results and discussion

### 3.1. Preparation of ACZ-loaded cubosomes

All the prepared ACZ-loaded cubosomal dispersions appeared as uniform opaque white mixtures without visible aggregates.

### 3.2. Characterization of ACZ-loaded cubosomes dispersions

#### 3.2.1. Particle size, PDI, and zeta potential

Particle size greatly influences the physical stability of cubosomes and their penetration through the corneal membrane and accordingly the ocular bioavailability of the loaded drug (Huang et al., 2017; Gaballa et al., 2020).

The mean particle size, polydispersity index, and zeta potential of the prepared cubosomes are listed in Table 3. It was found that variation in formulation factors, namely, oil: water ratio (lipid phase concentration), GMO: P 407 ratio, type, and concentration of water stabilizer had a significant effect on the particle size of the formed cubosomes (*p* < .0001, by applying one way ANOVA using GraphPad Instat. Software).

**Table 3.** Characterization parameters values for ACZ loaded cubosomes (mean ± *SD*, *n* = 3).

Formulation	Particle size (nm)	Polydispersity index (PDI)	Zeta potential (mV)	Entrapment efficiency (E.E) (%)	pH
F1	536.4 ± 3.1	0.24 ± 0.03	−11.8 ± 1.5	48.6 ± 0.78	7.1
F2	953.8 ± 4.2	…	…	…	…
F3	1070 ± 2.2	…	…	…	…
F4	610.9 ± 3.4	0.35 ± 0.05	−11.2 ± 2.8	40 ± 0.89	7
F5	359.5 ± 2.8	0.18 ± 0.03	−10.8 ± 3.2	59.8 ± 0.82	7.2
F6	537.3 ± 4.4	0.22 ± 0.04	−10.1 ± 2.5	36.6 ± 1.12	7.4
F7	433.7 ± 2.9	0.33 ± 0.06	−13.7 ± 2.1	51 ± 1.81	7
F8	560 ± 1.3	0.29 ± 0.03	−12.9 ± 1.8	25.3 ± 0.87	7.2

**Figure 1.**
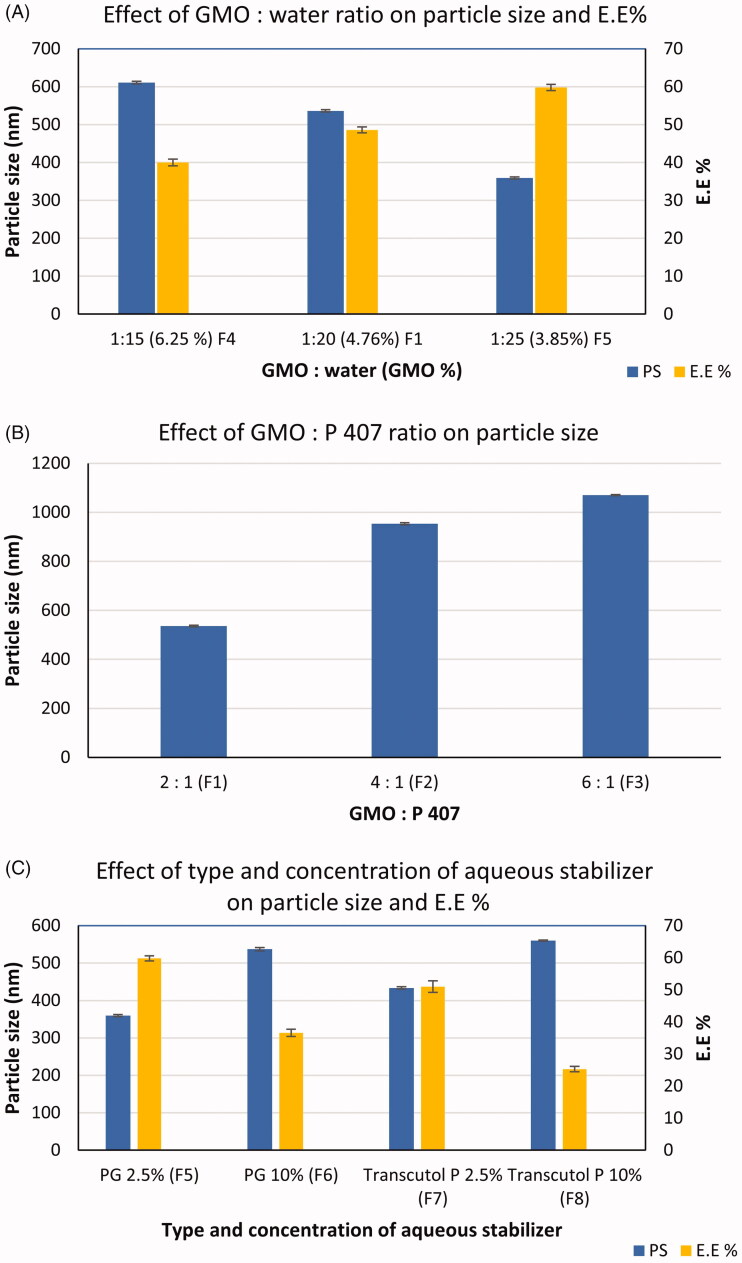
Effect of formulation variables on particle size and E.E% (mean ± *SD*) of cubosomes (*n* = 3). (a) effect of GMO : water ratio, (b) effect of GMO: P 407 ratio, (c) effect of type and concentration of aqueous stabilizer.

The effect of the oil: water ratio was studied by comparing F4, F1, and F5 in which the ratio was 1:15, 1:20, and 1:25, respectively. It was found that the size of the particles decreases significantly (*p* < .001) as the percentage of oil decreases compared to water as shown in Figure 1(a). The increase in the concentration of lipid phase from 3.85% in F5 to 6.25% in F4 may probably result in elevation of dispersion viscosity that may hinder the breaking down of the formed bicontinuous structures into small-sized ones (Elgindy et al., 2016). The obtained result was similar to that found by Eldeeb et al. in formulation and evaluation of cubosomes drug delivery system for treatment of glaucoma (Eldeeb et al., 2019).

In addition, by comparing F1, F2, and F3 which were prepared using ascending values of GMO: P407 ratio, it was clear that as that ratio increases from 2:1 to 6:1, significantly larger particle size is obtained (*p* < .001), as shown in Figure 1(b). This observation could be explained by the importance of P407 in the reduction of interfacial tension between the oil droplets and the aqueous phase, thus allowing the formation of homogenous and uniform emulsions with smaller droplet sizes. This was in agreement with the observation of Elgindy et al in the formulation of self-assembled nano-architecture liquid crystalline particles as a promising carrier for progesterone transdermal delivery (Elgindy et al., 2016). It could be noticed that F2 and F3 had a large particle size of 953.8 ± 4.2 and 1070 ± 2.2, respectively, and therefore would be excluded from further studies.

Also, by studying the effect of the concentration of the aqueous phase stabilizers (PG or Transcutol^®^ P), it was found that increasing its concentration led to a significant increase in the particle size (*p* < .001) from 359.5 ± 2.8 (F5) to 537.3 ± 4.4 (F6) when PG was used and from 433.7 ± 2.9 (F7) to 560 ± 1.3 (F8) in case of using Transcutol^®^ P, as represented in Figure 1(c). This result may be due to their association along the GMO-water interface to act as a steric barrier to prevent particles aggregation. The deposition of excess amount in case of higher concentrations on the surface of cubosomes led to an increase in the size of the particles.

Furthermore, changing the type of aqueous phase stabilizer from PG to Transcutol^®^ P resulted in a significant increase in particle size (*p* < .001) at both used concentrations (2.5 and 10%), Figure 1(c). This could be attributed to the structure difference between the two molecules as PG possess a smaller molecule than Transcutol^®^ P (three-carbon chain length for PG *vs.* six carbons in Transcutol^®^ P).

PDI values provided estimation about the homogeneity of particles and the width of particle size distribution. As shown in Table 3, all formulations showed PDI values ranged from 0.18 ± 0.03 to 0.35 ± 0.05 which indicated homogenous particle size distribution.

It is clear that all of the formulations have negatively charged zeta potential which may be attributed to the ionization of the free oleic acid that present in the monooleate (anionic) that makes the dispersion anionic in nature (Svensson et al., 2008; Hundekar et al., 2014; Mohyeldin et al., 2016). In addition to the presence of P 407 that interact by hydroxyl ions with the aqueous medium (Rizwan et al., 2009).

It was previously reported that the particles with negative charge could permeate through the skin via channels created by the repulsive forces between negatively charged skin lipids and particles (Kohli & Alpar, 2004).

Evaluation of the zeta potential of the prepared cubosomal dispersion is important to ensure the long-term stability of the colloidal dispersion. Although the values of zeta potential of all formulations were not high enough (between −10.1 and −13.7 mv) to avoid the aggregation of particles by electric repulsive effect, the presence of P 407, would stabilize the cubosomes by the adsorption of its polypropylene oxide copolymer (hydrophobic moieties) on the outer surface of the cubosomes that lead to shielding of the inverted-type self-assembled lipid nanoparticles from the surrounding aqueous medium, whereas the polyethylene oxide copolymer (the hydrophilic moieties) will suspend in water (Larsson, 1989; Gustafsson et al., 1996; Gustafsson et al., 1997).

#### 3.2.2. Entrapment efficiency (E.E%)

The E.E % of ACZ in the prepared cubosomes is presented in Table 3. All of the formulations showed E.E % ranged from 25.3 ± 0.87 to 59.8 ± 0.82%. Low E.E % of ACZ was observed for the prepared formulations which could be explained in the lights of ACZ solubility. ACZ is a poorly soluble drug in both water and oil (Barar et al., 2008) and its low oil solubility may be the reason for its low E.E%.

It could be noticed that formulation factors that increase ACZ solubility in the aqueous phase had a negative effect on drug E.E%, namely; type and concentration of aqueous stabilizer and oil: water ratio. It was clear that the use of Transcutol^®^ P caused a significant decrease in drug E.E% (*p* < .001) when compared with PG at the level of the two concentrations used. This could be attributed to higher ACZ solubility in Transcutol^®^ P than in PG (Morsi et al., 2014). In addition, increasing the concentration of the aqueous stabilizer from 2.5% to 10% caused an increase in aqueous solubility of ACZ and a consequently significant decrease (*p* < .001) in its E.E% from 59.8 to 36.6 for PG and from 51 to 25.3% in case of Transcutol^®^ P as shown in Figure 1(c).

Furthermore, oil: water ratio had a negative effect on E.E%; increasing this ratio by decreasing aqueous proportion in formulation led to a significant decrease (*p* < .001) in E.E% of ACZ as in F5 (1:25), F1 (1:20), and F4 (1:15) as represented in Figure 1(b). This finding was in contrast to the results obtained by Younes et al in preparation of corneal targeted sertaconazole nitrate-loaded cubosomes (Younes et al., 2018). As the PG is mainly distributed in the aqueous phase, its concentration within the aqueous phase decreased by increasing the proportion of water which resulted in decreasing in water solubility of ACZ and consequently improving its E.E%.

#### 3.2.3. Determination of pH values

However, the eye can tolerate a pH range between 4.5 and 11.5 due to the buffering action of the tears, the pH in the range of 7.2 ± 0.2 is considered ideal for maximum comfort (Shanmugam et al., 2011). All of the prepared formulations of cubosomes showed pH values between 7 and 7.4 which are in the normal pH range of the physiological pH of the eye. Therefore, no ocular irritation may occur due to pH.

### 3.3. Ex vivo corneal permeation studies

**Figure 2.**
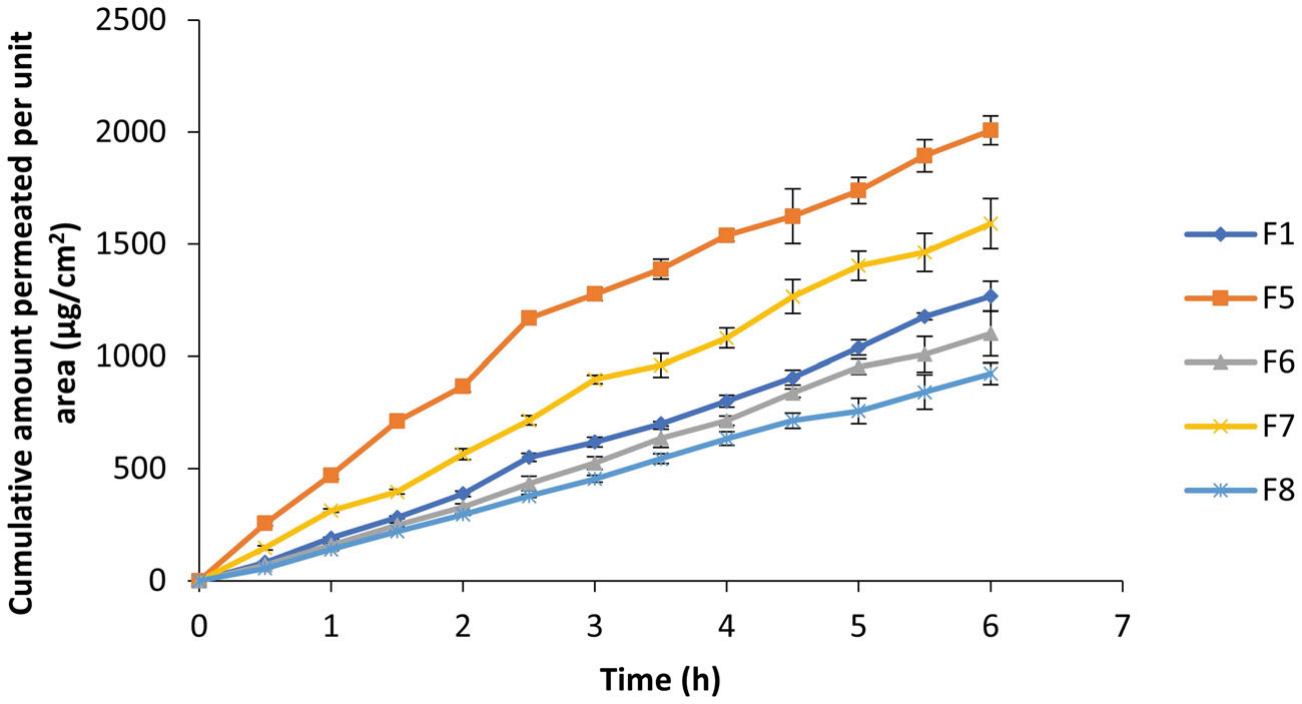
ACZ permeation from different ACZ-loaded cubosomes through the excised goat cornea (mean ± *SD*, *n* = 3).

The poor solubility of ACZ in both lipid and water limits its transfer through the corneal epithelium and endothelium, and also the transfer through the hydrophilic stroma, respectively (Barar et al., 2008). The amounts of ACZ permeated per unit area through the excised cornea at different times from different formulations are represented graphically in Figure 2.

The results of the present study showed that there was no lag time in ACZ permeation across corneas from the tested formulations. This may be due to the permeation-enhancing effect of both GMO and P407.

ACZ permeation through goat corneas was characterized by two phases. In the first 2–3 h the penetration of ACZ was fast followed by a slower drug penetration phase. The fast ACZ permeation through the corneal tissue may be due to the similarities between the cubosome as bicontinuous lipid bilayer structure, and that of the cell membranes of corneal epithelium. This similarity eases membrane fusion and therefore allowed the direct passage of ACZ into corneal cells (Gan et al., 2013; Chen et al., 2014). The decrease in the permeation of ACZ in the second phase may be attributed to the ability of cubosomes to form a depot in the lipid part of the corneal cells (Esposito et al., 2005).

Comparing cumulative amount of ACZ permeated after 6 h from F1 and F5, it was found that despite the decrease in the oily phase content from 4.76 to 3.85% w/w and therefore the amount of permeation enhancers (GMO and P 407), the amount of ACZ permeated was increased significantly (*p* < .001). This could be due to the increase in particle size and viscosity of formulation which accompanied the increase in the percentage of oil.

On the other side, it was found that increasing % PG in formulation from 2.5% (F5) to 10% (F6) resulted in a significant decrease in drug permeation (*p* < .001). This could be attributed to the increase in particle size by increasing PG. Also, F6 showed lower E.E% than F5 which may be another reason for the decrease in ACZ permeation.

The permeation parameters of ACZ in the different formulations were shown in Table 4. Analysis of data from Table 4 indicated that F5 was the most efficient in drug permeation according to the cumulative amount permeated per unit area, steady-state flux, and permeability coefficient values. A four-folds increase in permeability coefficient was obtained in the case of F5 when compared with the permeability coefficient of ACZ stated in the literature (Granero et al., 2008).

**Table 4.** Permeation parameters of ACZ from various formulations across goat cornea.

Formulation	Amount of ACZ permeated/ unit area (Q/A) after 6 h (µg·cm^−2^)	Lag time (min)	Steady state permeation (J) (µg·cm^−2^·h^−1^)	Permeability coefficient (cm·s^−1^)
F1	1267.1 ± 67.1	0	204.2	7.09 × 10^−6^
F5	2007.3 ± 64.3	0	459.8	1.597 × 10^−5^
F6	1101.5 ± 100.7	0	167.8	5.826 × 10^−6^
F7	1591 ± 111.4	0	283.6	9.847 × 10^−6^
F8	923.2 ± 48.4	0	147.7	5.128 × 10^−6^

### 3.4. Corneal hydration

The hydration level of the normal cornea was reported to be 76–80% (Huang et al., 2017). Any change in this level may harm the endothelium or the epithelium. Corneal hydration of the excised corneas exposed *in-vitro* to the different cubosomal formulations were within the normal hydration levels, which means that these formulations did not cause any damage to the corneal tissues.

### 3.5. Selection of the optimum formulation

**Figure 3.**
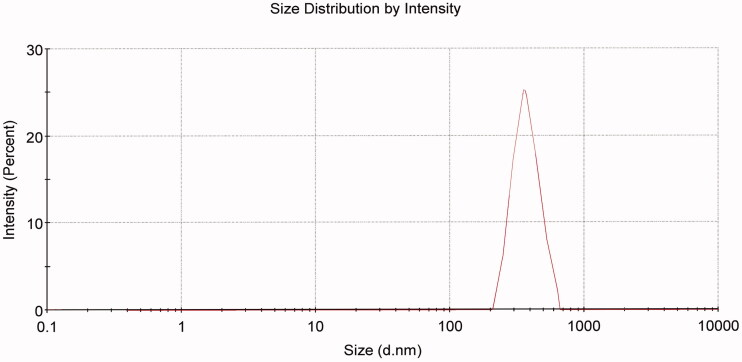
Particle size distribution of F5 cubosomes dispersion.

F5 was selected for further *in-vivo* studies as it showed the smallest size of particles with homogenous distribution as shown in Figure 3 and the highest E.E% among other tested formulations. It also showed superior enhancement in ACZ permeation through excised corneas.

### 3.6. Morphology of cubosomes

**Figure 4.**
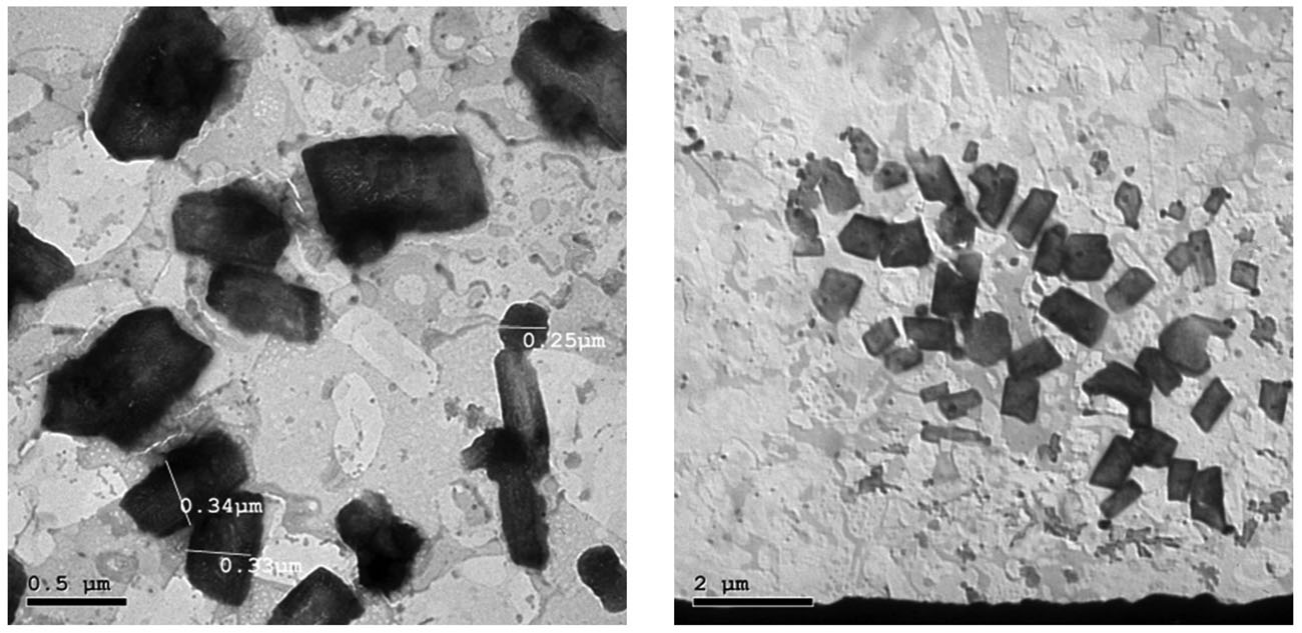
Transmission electron microscopy images of ACZ-loaded cubosomes.

TEM images of the selected ACZ-loaded cubosomes (F5) revealed that the prepared cubosomes are cubic in shape and well-separated from each other without aggregations, as shown in Figure 4. Furthermore, the particle size detected in Figure 4 was around 300 nm which is consistent with that determined by dynamic light scattering.

### 3.7. Effect of gamma sterilization

Sterilization of ophthalmic products is a necessary process to ensure the absence of micro-organisms in the formulation. Sterilization by filtration technique using 0.22 µm membrane filters was not suitable for our formulation because of particle size. Therefore, the terminal Gamma sterilization technique was selected. Particle size, PDI, zeta potential, and E.E% values of F5 after sterilization did not differ significantly (*p* > .01) from those measured before sterilization.

### 3.8. Ocular irritation studies

The irritation study did not result in any alteration in the conjunctiva or other parts of the rabbit’s eye. After instillation of F5, no significant conjunctival discharge, redness, or chemosis were detected in any of the rabbits except slight redness and lachrymation was observed in one of the rabbits in the first 30 min. Therefore, F5 can be considered as a non-irritant formulation to the eye.

The results of *in-vivo* ocular irritation test are represented in Table 5 where the overall ocular irritation index was calculated for each time point after instillation of F5 and normal saline solution (0.9% NaCl).

**Table 5.** Ocular irritation scores after instillation of F5 and 0.9 N NaCl.

Time (min)	5	10	15	30	60	120
F5	0.33	0.33	0.67	0.67	0	0
0.9 N NaCl	0	0	0	0	0	0

### 3.9. Therapeutic efficiency study

**Figure 5.**
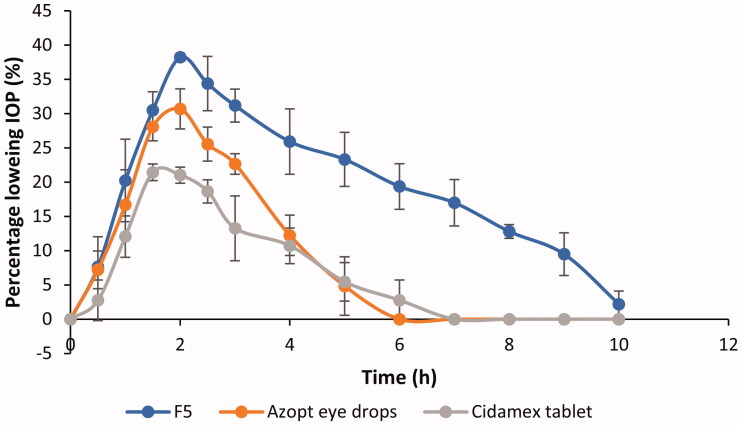
Percentage decrease in IOP after topical application of F5, Azopt eye drops, and oral administration of Cidamex tablets (mean ± *SD*, *n* = 3).

The IOP lowering activity profiles of F5, Azopt^®^ eye drops, and Cidamex^®^ tablets were represented in Figure 5 and the calculated pharmacodynamic parameters were listed in Table 6.

**Table 6.** The calculated pharmacodynamic parameters of F5, Azopt eye drops, and Cidamex tablets (mean ± *SD*, *n* = 3).

Formulation	Max. % decrease in IOP	*T*_max_ (h)	AUC_0–10_	MRT (h)
F5	38.22 ± 0.54	2 ± 0	196.9 ± 7.88	4.22 ± 0.17
Azopt eye drops	31.14 ± 2.61	1.83 ± 0.29	84.06 ± 5.37	2.36 ± 0.22
Cidamex tablets	21.99 ± 0.99	1.67 ± 0.29	64.5 ± 13.65	2.62 ± 0.16

As ACZ is not available as a topical formulation in either the local or the international market, Azopt^®^ eye drop was selected to be the topical control in this study. Azopt^®^ was selected as it contains brinzolamide which is intrinsically more permeable than ACZ and presents in higher concentration (1%) in comparison to 0.8% ACZ in the tested formulation (F5) which makes the comparison an interesting challenge. Cidamex^®^ tablets were selected as the second control to emphasize the difference in the therapeutic efficacy between the optimized topical ACZ cubosomal dispersion (F5) and the oral tablets in dose 9 mg (the calculated dose of rabbits of average weight 2.5 kg).

Comparing AUC_0–10_ values as an indicator of ocular bioavailability, it is evident that the value of this parameter was ranked in the following order F5 > Azopt^®^ > Cidamex^®^. By applying ANOVA statistical test, AUC_0–10_ of F5 was significantly higher than that of Azopt^®^ and Cidamex^®^ by 2.3 and 3 times, respectively (*p* < .001).

Azopt^®^ eye drops showed a significantly lower therapeutic efficacy when compared with the ACZ-loaded cubosome nano-dispersion and this may be attributed to the relatively fast clearance of traditional eye drops (Azopt^®^) from the pre-ocular surface. In addition, Cidamex^®^ tablets showed a decrease in its ocular bioavailability which may be due to the non-selective inhibition of carbonic anhydrase enzyme by ACZ after oral administration with taking into consideration that the administered tablet dose was equivalent to 9 mg ACZ while the dose of ACZ in 50 µl F5 was 0.4 mg only.

Regarding the duration of IOP reduction, it is clear from Figure 5 that F5 showed a prolonged effect compared to Azopt^®^ or Cidamex^®^. This result was consistent with the calculated MRT as F5 had an MRT 1.8 and 1.6 times higher than those of Azopt^®^ and Cidamex^®^, respectively.

Higher therapeutic efficacy as well as the sustained effect observed in F5 may result from the increase in corneal permeation and ocular residence time by the effect of cubosomes. The higher corneal permeation is probably due to the permeation enhancing effect of both GMO and P407 and structural similarities between the bicontinuous lipid bilayer of cubosomes and cell membranes of corneal epithelium. This similarity facilitates membrane fusion and therefore allowed the direct passage of ACZ into corneal cells (Gan et al., 2013; Chen et al., 2014).

F5 showed a prolonged residence time which is supposed to be due to the bioadhesive properties of the lipid bilayer forming cubosomes, and the non-specific interaction (hydrophobic and Van der Waals) of cubosomes with the superficial oily layer of the tear film (Verma & Ahuja, 2016). Furthermore, the cubosomes can form a depot in the lipid part of the corneal cells (Esposito et al., 2005).

It was found that the untreated eye did not have a change in IOP during the experiment which indicates that F5 showed the inhibition effect of carbonic anhydrase enzyme only within the treated eye and the detected IOP lowering effect is not because of any systemic absorption followed by redistribution.

## 4. Conclusion

ACZ-loaded cubosomes were successfully prepared and optimized via different characterization techniques as well as *ex-vivo* permeation. The optimized formulation (F5) showed a particle size of 359.5 ± 2.8 nm, polydispersity index 0.18 ± 0.03, and zeta potential −10.8 ± 3.2 mV and it showed 4-folds enhancement in permeability coefficient of ACZ when compared with that stated in the literature. Moreover, F5 was found to be non-irritant to rabbits’ eyes when evaluated by the modified Draize test. Furthermore, F5 showed higher therapeutic efficacy than both Azopt^®^ eye drops and Cidamex^®^ tablets as confirmed by its enhanced ocular bioavailability represented by AUC_0–10_ as well as prolonged effect represented by MRT. Therefore, ACZ-loaded cubosomes provide a novel topical drug delivery system for glaucoma management with excellent IOP lowering activity extended for more than 9 h accompanied by a tremendous decrease in the used dose. Additionally, this novel drug delivery system offers a targeted inhibition of carbonic anhydrase enzyme present in the eye which led to the great reduction or disappearance of severe systemic side effects of the marketed drug product, Cidamex^®^ tablets.
